# Cellular crosstalk in fibrosis: Insights into macrophage and fibroblast dynamics

**DOI:** 10.1016/j.jbc.2025.110203

**Published:** 2025-05-05

**Authors:** Zachary S.C.S. Froom, Neal I. Callaghan, Locke Davenport Huyer

**Affiliations:** 1 School of Biomedical Engineering, Faculties of Medicine and Engineering, Dalhousie University, Halifax, Nova Scotia, Canada; 2 Department of Medicine, Dalhousie University, Halifax, Nova Scotia, Canada; 3 Department of Microbiology & Immunology, Faculty of Medicine, Dalhousie University, Halifax, Nova Scotia, Canada; 4 Department of Biomaterials & Applied Oral Sciences, Faculty of Dentistry, Dalhousie University, Halifax, Nova Scotia, Canada; 5 Division of Plastic Surgery, Nova Scotia Health, Halifax, Nova Scotia, Canada

**Keywords:** fibrosis, inflammation, macrophages, fibroblasts, extracellular matrix

## Abstract

Pathological fibrosis, the excessive deposition of extracellular matrix and tissue stiffening that causes progressive organ dysfunction, underlies diverse chronic diseases. The fibrotic microenvironment is driven by the dynamic microenvironmental interaction between various cell types; macrophages and fibroblasts play central roles in fibrotic disease initiation, maintenance, and progression. Macrophage functional plasticity to microenvironmental stimuli modulates fibroblast functionality by releasing pro-inflammatory cytokines, growth factors, and matrix remodeling enzymes that promote fibroblast proliferation, activation, and differentiation into myofibroblasts. Activated fibroblasts and myofibroblasts serve as the fibrotic effector cells, secreting extracellular matrix components and initiating microenvironmental contracture. Fibroblasts also modulate macrophage function by releasing their own pro-inflammatory cytokines and growth factors, creating bidirectional crosstalk that reinforces the chronic fibrotic cycle. The intricate interplay between macrophages and fibroblasts, including their secretomes and signaling interactions, leads to tissue damage and pathological loss of tissue function. In this review, we examine macrophage–fibroblast reciprocal dynamic interactions in pathological fibrotic conditions. We discuss the specific lineages and functionality of macrophages and fibroblasts implicated in fibrotic progression, with focus on their signal transduction pathways and secretory signaling that enables their pro-fibrotic behavior. We then finish with a set of recommendations for future experimentation to develop a set of potential targets for anti-fibrotic therapeutic candidates. Understanding the cellular interactions between macrophages and fibroblasts provides valuable insights into potential therapeutic strategies to mitigate fibrotic disease progression.

Fibrosis is characterized by the deposition of fibrous connective tissue in response to an acute insult, such as physical trauma or inflammation, to render mechanical stability or fill space in a wound environment. Pro-fibrotic signaling can allow for the formation of a protective capsule around pathogens (*e.g.*, tuberculosis, parasites, indolent microbial colonization) to limit their spread (1), following Th2 or highly inflammatory Th17 signaling. Fibrotic tissue can also aid in the integration of biomedical implants, helping to adhere the implant into the tissue (2). While largely adaptive, fibrotic responses can become pathological. Pathological fibrosis is a spatiotemporal disease that is characterized by the excessive formation and replacement of functional native tissues with fibrotic connective tissue through excessive extracellular matrix (ECM) deposition that that then limits further remodeling into functional tissue (3, 4). This leads to tissue stiffening and thickening that subsequently impairs native tissue function (3, 4, 5). Fibrosis presents in many chronic syndromes localized to specific tissue microenvironments of the human body; for example, dysregulated development of fibrotic tissue can affect the heart (6, 7, 8, 9), lungs (10, 11, 12, 13, 14), liver (15, 16, 17), kidney (18, 19, 20, 21), and skin (22, 23). Patients with fibrotic disease typically experience chronic pain and fatigue and may exhibit serious organ-specific complications (3, 24, 25) that can considerably lower their quality of life (3, 5, 24). As such, pathological fibrosis significantly contributes to worldwide morbidity and mortality (3); it is estimated that fibrotic conditions impact 10% of the global population (26) and can be attributed to approximately 35% to 45% of annual global deaths (4, 27).

Fibrotic conditions primarily arise as complications of chronic inflammatory or autoimmune conditions, such as systemic connective tissue disorders or rheumatic diseases (23, 28, 29). This frequently results in late diagnoses of fibrotic conditions, and corresponding delay in intervention until there is irreversible loss of native tissue function and organ damage (30). The lack of specific non-invasive biomarkers further exacerbates the potential for early diagnosis (30), frequently necessitating invasive techniques (*i.e.* biopsies) to positively identify disease at more advanced stages of fibrotic progression (31, 32). Once diagnosed, pathological fibrotic conditions are difficult to treat due to their multivariate etiology and patient-specific heterogeneity. This regularly results in the targeting of single biological pathways, which often proves ineffective in halting or counteracting fibrotic progression (3, 4). This complicates efforts to identify therapeutic targets and extends the time needed for research of new anti-fibrotic therapies (4, 33, 34). As such, there are very few FDA-approved therapies that target pathological fibrosis directly (35).

Across fibrotic conditions, chronic tissue inflammation causes pathological outcomes by establishing cycles of mutually reinforcing inflammation and fibrosis. This loop is generated due to the failure to clear innate inflammatory cell populations from the disease microenvironment, subsequently establishing a population of chronically activated pro-fibrotic cells (4, 5, 36). As fibrosis is contained in its localized microenvironment, this inflammatory loop is self-amplifying and persistent. Central to the pathogenesis of fibrosis is the macrophage-fibroblast axis, which orchestrates the chronic inflammatory and pro-fibrotic signaling that drives excessive ECM deposition (3, 4, 5, 37, 38, 39, 40). Persistent inflammatory signaling seen in fibrotic conditions is primarily perpetuated by chronically activated macrophages (39, 41). In addition to self-amplifying pro-inflammatory signaling, macrophages play a key role in the pro-fibrotic signaling to fibroblast cells (37, 38, 39, 40) that establishes a population of chronically activated ECM-producing fibroblasts and myofibroblasts (4, 5, 38). The continual production of ECM overwhelms the myofibroblast population, leading to improper matrix remodeling (37, 40, 42) and excessive production that causes a variety of clinical complications.

In this review, we seek to elucidate the pro-fibrotic macrophage-fibroblast axis and their reciprocal dynamic interactions that persist across clinical presentations of fibrosis. We discuss the specific linages and functionality of macrophage and fibroblasts implicated in fibrotic disease progression, with focus on the key signal transduction pathways and secretory signaling that enables their pro-fibrotic functionality. This is followed by a detailed discussion of how reciprocal signaling vary according to tissue environment, focusing on the major fibrotic pathologies and the subsequent downstream effects of fibrosis. We conclude with a discussion of recommendations for future experimentation to identify new anti-fibrotic therapies and biomarkers for earlier and more accurate diagnosis.

## Macrophage lineages in fibrotic diseases

At the center of the pathogenesis of fibrotic conditions is the macrophage, a highly diverse and versatile innate immune cell (39, 41). Macrophages have remarkable adaptability, allowing dynamic responses to specific tissue microenvironmental cues (43, 44) and subsequent functional roles that range from pro-inflammatory to pro-reparative activities that are crucial to tissue homeostasis (41, 43, 45, 46). Pro-inflammatory functionality allows macrophages to combat pathogens and damaged cells, as well as the initiation of pro-reparative activities upon clearance of the acute insult (41, 46). Pro-reparative macrophage functionality achieves controlled resolution of inflammation signaling, clearance of cellular and tissue debris generated by the initial insult or the inflammatory efforts, and tissue regeneration *via* signaling to stromal cells to deposit new ECM (45, 46). These functional roles are shaped by the heterogeneity and plasticity of macrophage populations (43, 47, 48, 49). In the fibrotic microenvironment, macrophage populations exist in both pro-inflammatory and pro-reparative functionalities, with populations shifting dynamically between them (47).

Macrophages are derived from both resident and infiltrating lineages (48, 49, 50, 51). Resident macrophages originate during development from embryonic progenitors, and their populations are maintained locally in their specific tissues by proliferation (48, 50, 51). Infiltrating macrophages are derived from circulating monocytes and are recruited to sites of tissue injury from chemotactic mediators released by resident stromal and macrophage populations acting with pro-inflammatory functionalities (41). Understanding the distinct roles of these populations and the complexity of macrophage functionality is crucial to understanding their contributions to fibrotic progression.

### Resident and infiltrating macrophages in the fibrotic microenvironment

There is significant overlap in the function of tissue resident and infiltrating macrophages, but they differ in how they contribute to fibrotic tissue development. Infiltrating monocytes/macrophages play prominent roles in fibrosis through their ability to vastly amplify the inflammatory response following tissue injury. After their initial recruitment by resident stromal cells and macrophages, infiltrating macrophages quickly overwhelm the resident populations, becoming the dominating macrophage population (52). Infiltrating monocytes/macrophages take on a pro-inflammatory role and, as the dominant population, they are largely responsible for establishing the chronically inflamed environment. This ultimately results in a pro-fibrotic immune cell persistence in the microenvironment due to the over-recruitment of infiltrating macrophages and a shift toward a more pro-fibrotic functionality, subsequently leading to the overproduction of pro-fibrotic factors and ECM proteins (39, 41).

In contrast, resident macrophages are highly specialized to their tissue environment, enabling them to perform roles critical to maintaining homeostasis (48, 51, 53). For example, lung alveolar macrophages (AMs) are indispensable for clearance of inhaled pathogens and particulate matter (54, 55, 56). Every tissue has its own tissue-resident macrophages; some examples of resident macrophage populations include lung interstitial macrophages (IMs) (57), Kupffer cells (KCs) of the liver (58, 59, 60, 61, 62), kidney resident macrophages (KRMs) (63, 64), and cardiac resident macrophages (CRMs) (65, 66, 67). In general, resident macrophages are involved in the acute establishment of the inflammatory environment that leads to the recruitment of infiltrating macrophages (52). As the fibrotic disease progresses, resident macrophages become chronically activated and subsequently release pro-fibrotic cytokines and growth factors, further adding to the fibrotic niche (39, 52).

Ongoing lineage tracing efforts continue to refine tissue-specific models of infiltrating and tissue-resident macrophage influences toward fibrotic progression. For example, infiltrating macrophages have been demonstrated as the primary source of the macrophage population in pulmonary fibrosis that drives fibroblast activation (68, 69). In cardiac fibrotic pathologies, cardiac resident macrophage populations secrete ECM proteins while remaining distinctive from fibroblast populations (70). While preliminary lineage tracing efforts have demonstrated that infiltrating macrophages are primarily driving fibrotic progress (69), tissue resident macrophages still maintain functionality in instructing fibrosis (70). The role in fibrotic progression that each type of tissue resident macrophage plays in their specific tissue environment is further discussed in Macrophage–fibroblast dynamics in late-stage pathologies.

### Macrophage functional states: The M1-M2 paradigm

**Figure 1:**
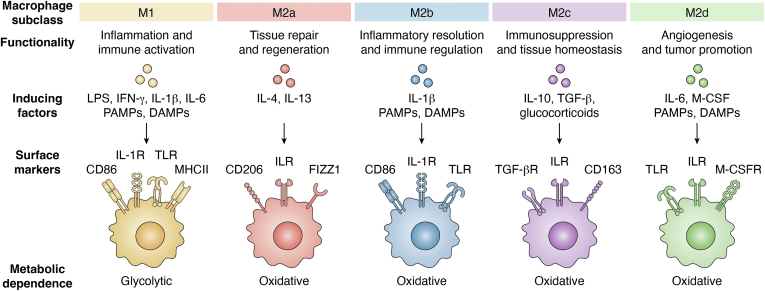
**Canonical M1-M2 macrophage paradigm.** Macrophage activity spans a wide range of functionality, which is typically stratified using the M1-M2 paradigm. Macrophage subclasses are defined by their *in vitro* inducing factors and, subsequently, what surface markers they express. M1 macrophages are dependent on glycolytic methods for energy production and exhibit pro-inflammatory functionality, while M2 macrophages typically are dependent on oxidative methods and exhibit more pro-reparative functionality.

Macrophage populations are highly plastic, capable of dynamically altering their functionality in response to local environmental cues (43, 44, 71, 72, 73). This plasticity is often conceptualized within the canonical M1-M2 paradigm, which describes macrophages along a spectrum of activity with ranging functionality from pro-inflammatory to pro-reparative, respectively (Fig. 1) (72, 74, 75, 76). The macrophage subsets along this canonical spectrum are defined based on their *in vitro* induction factors as well as the expression of key surface markers that highlight the breadth of macrophage functionality. The M1-M2 paradigm falls short in the classification of complex and unique *in vivo* tissue or disease environments, such as fibrotic pathologies, due to oversimplification, but provides a necessary appreciation of the extremes in macrophage functional polarity. The interplay and dynamic functionality shifting of macrophage populations is what allows them to be a conserved, significant effector cell across all fibrotic pathologies.

#### M1 macrophages

M1 (classically activated) macrophages are induced by pathogen-associated molecular patterns (PAMPs), such as lipopolysaccharide (LPS), and/or cytokines, such as interferon-γ (IFN-γ), in either a single or co-stimulatory fashion (72, 76). Outside these canonical stimuli, M1 macrophages can also be induced by a variety of pattern recognition receptor (PRR) stimuli, such as damage-associated molecular patterns (DAMPs), and other cytokines, including interleukin-1β (IL-1β) and IL-6 (73, 77). M1 macrophages are characterized by the surface expression of cluster of differentiation (CD)-86 and major histocompatibility complex class II (MHC-II), as well as their pro-inflammatory and pathogen clearance functions (*via* phagocytosis) through the production of pro-inflammatory cytokines, including tumor necrosis factor-α (TNF-α), IL-1β, and IL-6, and reactive oxygen/nitrogen species (ROS/RNS). Additionally, M1 macrophages are responsible for the activation of the adaptive immune system *via* antigen presentation by MHC-II (73, 74, 76).

M1 macrophages primarily rely on glycolysis as a catabolic source, with two distinctive breaks in their tricarboxylic acid (TCA) cycle (78, 79). The enzyme isocitrate dehydrogenase (IDH) is inhibited by autocrine signaling with IFNs (80), leading to the accumulation of itaconate through shunting with aconitate decarboxylase 1 (ACOD1) (81, 82). Accumulated itaconate inhibits succinate dehydrogenase (SDH) and causes succinate accumulation (83, 84). Elevated succinate levels enable reverse electron transport (RET) to produce ROS/RNS *via* succinate oxidation (85, 86, 87). Additionally, succinate accumulation stabilizes HIF-1α, which promotes IL-1β maturation through activation of the NLRP3 inflammasome (87, 88, 89).

#### M2 macrophages

M2 (alternatively activated) macrophages are associated with tissue repair, inflammatory resolution, and ECM deposition through the production of anti-inflammatory cytokines, such as IL-10, and growth factors such as transforming growth factor-β (TGF-β) (73, 76). M2 macrophages are further subdivided into subclasses defined by *in vitro* inducing factors, most notably M2a, M2b, M2c, and M2d (74, 76, 90, 91). While all M2 subclasses may be found in an incipient pro-fibrotic functionality, M2a and M2c macrophages have been particularly implicated in the progression of fibrosis (92). M2a macrophages have demonstrated roles in enhanced ECM deposition and activation of fibroblasts (*i.e.*, growth factor signaling, such as TGF-β secretion) (92, 93, 94, 95). This subclass is induced by the stimulus of cytokine receptors by IL-4 and/or IL-13 and is characterized by the expression of CD206 (mannose receptor) and found in inflammatory zone 1 (FIZZ1) (93, 94, 95). Conversely, M2c macrophages have been implicated in promoting ECM remodeling (*i.e.*, matrix metalloproteinases (MMP) and tissue inhibitors of metalloproteinases (TIMPs) secretion) and reduction of inflammatory signaling (*e.g.*, through IL-10 signaling) (76, 92, 95, 96). This subclass is induced by IL-10, TGF-β, and glucocorticoids and expresses CD163 (hemoglobin scavenger receptor). Other M2 subclasses (*i.e.* M2b and M2d) are less implicated in fibrotic disease. M2b macrophages are induced by toll-like receptor (TLR) and IL-1R agonists, express canonical M1-like surface expression markers (*e.g.*, CD86), and contribute to the resolution of inflammation (76, 97). Lastly, M2d macrophages are induced by TLR agonists such as IL-6 and macrophage colony-stimulating factor (M-CSF). These macrophages exhibit pro-angiogenic functions and are commonly associated with tumor microenvironments (98, 99, 100). M2 macrophages primarily rely on oxidative pathways as their source for energy production. Through the upregulation of fatty acid oxidation (FAO) and oxidative phosphorylation (OXPHOS), M2 macrophages can redirect the use of their glycolytic enzymes for anabolic processes such as ECM production (78, 79, 101).

### Limitations of the M1-M2 paradigm

While the M1-M2 binary classification system provides a useful framework for understanding the ranges of macrophage functionality, it does not fully capture the full breadth of function seen in pathological fibrosis. The tissue microenvironment profoundly influences macrophage functionality, blurring the distinction between subpopulations (94, 102). There is a significant degree of overlap in the markers and stimulatory agents that have been used to canonically define macrophages subclasses, complicating their identification (103, 104, 105, 106). For example, CD206 and CD163, commonly used to distinguish M2a and M2c macrophages, are often co-expressed in many tissue-specific contexts (95). This ambiguity has led to the use of relative expression profiles, such as high or low levels of these markers, to characterize distinctive subpopulations (102). Discrepancies in these classifications have become more apparent with the rise of scRNAseq methodology and datasets that allow for function-specific insight into macrophages in tissue environments of interest (107, 108, 109).

The metabolic states of macrophages in the M1-M2 paradigm are traditionally constrained to the notion that M1 macrophages are glycolytic and M2 macrophages are oxidative (79, 101). The dynamic nature of macrophages’ functionality is similarly echoed in their metabolic plasticity. The metabolic profile of macrophages is pivotal to shaping their functionality, particularly within the fibrotic microenvironment (79, 101). Hypoxia, a hallmark of fibrotic tissue, and the presence of oxidative stress (*e.g.*, ROS/RNS) significantly influence macrophage polarization through the stabilization of STAT6, modulating IL-4/IL-13 stimulation, and HIF-2α, promoting pro-reparative macrophage activation (110, 111, 112). In an attempt to remedy the hypoxic microenvironment, pro-reparative macrophages secrete pro-angiogenic factors, such as vascular endothelial growth factors (VEGFs) (113, 114). Under oxidative stress, resultant limitation of electron transport chain flux, M2 macrophages have been observed to shift toward the use of glycolysis as their primary energy source (115). Recognizing the intricacy of macrophage functionality is critical to parsing out the complexity of fibrotic pathophysiology.

## Macrophage signaling is central to fibrotic progression

Fibrotic progression is intricately modulated by macrophage signaling, which in turn governs the diverse roles of macrophages, from perpetuating the chronically activated environment to instructing the excessive deposition of ECM. The plasticity of macrophage populations allows transitions between pro-inflammatory and pro-reparative functionality to dynamically respond to the evolving fibrotic microenvironment. Early in fibrotic disease progression, macrophages predominantly adopt a pro-inflammatory functionality to establish the inflammatory niche and recruit additional immune cells. As the disease progresses, unresolved inflammation shifts macrophage populations toward pro-reparative functionality, where their excessive ECM synthesis and remodeling exacerbate fibrosis (44, 76).

While pro-inflammatory functionalities dominate early pro-fibrotic macrophage populations, the microenvironment actively instructs macrophage populations toward a pro-reparative, pro-fibrotic functionality at all stages following the initial pro-inflammatory insult (39). The signals that drive this switch increase over time, progressively leading to pro-reparative macrophages becoming the dominant population (39, 52). In the early inflammatory response to insult, the accumulation of apoptotic neutrophils and dying native cells initiates this functional shift, as their efferocytosis promotes anti-inflammatory and reparative programming in macrophages (116, 117). Simultaneously, early deposition of provisional ECM components begins to generate an inadequately perfused microenvironment and an associated onset of localized hypoxia that favor pro-reparative macrophage persistence (111, 118, 119). Provisional ECM also acts as an activating scaffold through cell-ECM contact-dependent signaling (*e.g.*, integrin αvβ3 binding to fibronectin) to increase pro-reparative functionality (120). Hypoxia associated pro-inflammatory macrophage ROS production further drives this shift through ROS-dependent stabilization of HIF-2α which subsequently increases pro-reparative functionality (119, 121, 122). As the response to the insult progresses, aberrant signaling and the shift to pro-reparative functionality leads to fibrosis development. The transition from pro-inflammatory to pro-reparative and now pro-fibrotic functionalities is further reinforced by sustained pro-fibrotic signaling from fibroblasts as well as from regulatory and Th2 T-cells, which continuously instruct macrophages to maintain pro-reparative functionality (123, 124, 125, 126). In parallel, the maturing and stiffening ECM and persistent metabolic stressors stabilize this functionality, ensuring that macrophages remain central to the dysregulation of ECM deposition and remodeling and fibrotic progression long after the initial inflammatory triggers have subsided (111, 127, 128).

The ability for macrophage populations to dynamically adapt to new environmental cues is reliant on their robust signal transduction pathways and subsequent secretome that define their signaling (44, 129). Ultimately, persistent macrophage activation leads to unrestrained signaling, subsequently leading to the dysregulation that drives fibrotic disease. Here, we define key macrophage signaling and its contribution to the dysregulated fibrotic macrophage pathology; this signaling further contributes to fibrotic progression through cellular crosstalk, which we discuss in Macrophage–fibroblast crosstalk in fibrotic microenvironments.

### Macrophage signal transduction pathways implicated in fibrotic progression

Macrophage functionality in fibrosis is determined by the dynamic interplay of receptor-mediated signaling and corresponding transcriptional regulation (44). These signal transduction pathways serve as the molecular switches that enable the necessary functional modularity of macrophages, ranging from establishing the pro-inflammatory microenvironment to instructing pro-fibrotic signaling (Table 1) (103). The complexity of the fibrotic microenvironment can be attributed to a high degree of overlap within these pathways that is exacerbated by chronic activation, which may modulate the intended functional changes into a pathological outcome (39, 94).

**Table 1:** Macrophage signal transduction pathways implicated in fibrotic progression

Pro-inflammatory			
Signalling pathway	Activating ligand	Function	Source(s)
NF-κB	LPS, TNF-α, IL-1β	Master transcriptional factor for pro-inflammatory function.	(130, 131, 132, 133)
STAT-1	IL-6, IFN-α, IFN-γ, EGF, PDGF	Regulates M1-like polarization and proliferation. Upregulates pro-inflammatory cytokine release.	(134, 135, 136)
HIF-1α	IL-1β, IL-6, IFN-γ, ROS/RNS	Stabilizes iNOS to upregulate ROS/RNS production. Facilities glycolytic dominance by upregulating PDK1 expression.	(121, 122, 137, 138, 139)

Pro-reparative			
Signalling pathway	Activating ligand	Function	Source(s)
STAT-6	IL-4, IL-13	Activates PPARδ/PPARγ and the expression of pro-fibrotic genes.	(148, 149, 150, 151, 152, 153, 154, 155)
PPARδ	STAT6	Inhibits NF- κB activity, induces antioxidant expression, and promotes FAO and mitochondrial biogenesis to promote anti-inflammatory functionality and OXPHOS	(163, 164, 165, 166, 167, 168)
PPARγ	STAT6	Inhibits NF- κB activity, induces the production of anti-inflammatory cytokines, and incudes mitochondrial biogenesis to promote anti-inflammatory functionality and OXPHOS.	(148, 150, 168, 169, 170, 171)
SMAD	TGF-β	Promotes the expression of ECM protein and remodeling genes. Facilities an autocrine activating loop through TGF-β	(156, 157, 158, 159, 160, 161, 162)

The main transduction pathways activated through inflammatory polarization are NF-κB STAT-1, and HIF-1α. NF-κB is induced by TLRs and IL-1R antagonists and acts as a master regulator of inflammation (130, 131, 132, 133). STAT1 is activated by IFN-γ through the JAK/STAT phosphorylation cascade; its activation promotes a pro-inflammatory functional state and accelerates proliferation (134, 135, 136). Finally, HIF-1α accumulates in response to pro-inflammatory signaling such as IFN-γ, which is later stabilized by succinate to upregulate ROS/RNS production that in turn promotes further induction of HIF-1α (121, 122, 137, 138, 139). Broadly, the modulation of these signal transducers leads to the upregulation of pro-inflammatory pathways, primarily pro-inflammatory cytokine and ROS/RNS production, and facilitates glycolytic reliance through the upregulation of glucose transporter 1 (GLUT1) (39, 101). Thus, the chronic activation of these pathways in a fibrotic microenvironment causes the over-production of inflammatory mediators, perpetuating pathology through the over-recruitment and activation of pro-fibrotic effector cells.

Macrophages polarized primarily through pro-reparative signaling (canonically IL-4 and IL-13) predominantly express their functionality through actions of STAT6 (140, 141, 142, 143). Both IL-4 and IL-13 act to phosphorylate STAT6 *via* JAK1/JAK3 or JAK2, respectively (144, 145, 146, 147). Consequently, STAT6 undergoes homodimerization to translocate to the nucleus and induce both PPARδ and PPARγ (148, 149, 150, 151). Nuclear translocation of STAT6 leads to subsequent cleavage to upregulate the expression of pro-fibrotic genes, including arginase-1 (ARG1), FIZZ1, and TGF-β (152, 153, 154, 155). The production of TGF-β facilitates an autocrine loop, wherein secreted TGF-β activates the SMAD transduction pathway (*i.e.* SMAD2 and SMAD3) (156, 157, 158, 159) that subsequently increases the expression of ECM protein and remodeling genes (157, 160, 161, 162). PPARδ promotes an anti-inflammatory functional state by decreasing the production of pro-inflammatory cytokines by inhibition of NF-κB and inducing the expression of antioxidant genes such as heme oxygenase-1 (HO-1) (163, 164, 165, 166). Additionally, PPARδ drives oxidative metabolism through promotion of FAO and mitochondrial biogenesis (167, 168). Like PPARδ, PPARγ dampens the production of pro-inflammatory cytokines by inhibiting NF-κB (150). PPARγ further promotes an anti-inflammatory functional state by increasing the production of anti-inflammatory cytokines, such as IL-10, stimulating mitochondrial biogenesis and boosting lipid metabolism through cholesterol efflux (148, 168, 169, 170, 171). By increasing cholesterol efflux, M2 macrophages decrease the likelihood of becoming highly inflammatory foam cells or lipid-laden macrophages (171, 172, 173, 174, 175). While these pathways appear to be inherently anti-fibrotic through the triggering of anti-inflammatory signaling and ECM remodeling pathways, when overactivated they may become pro-fibrotic primarily due to the over-production of ECM proteins (41, 176).

### Macrophage secretomes central to fibrotic progression

Macrophages exert their influence on fibrosis through a diverse array of secreted factors, collectively known as the secretome. The secretome of macrophages includes cytokines, chemokines, and MMPs that mediate autocrine and paracrine signaling, which shape cell behavior within the fibrotic niche (Table 2). The macrophage secretome is a continuum of their functional responses to activation signals, which are tightly regulated by transcriptional pathways such as NF-κB, STAT6, and SMAD signaling (130, 152, 157). The interplay between the secretomes of macrophages responding to either pro-inflammation or pro-reparative activation further underscores the macrophage population's plasticity and capacity to adapt to environmental cues. In fibrosis, these secretomes are dysregulated and temporally sustained through positive feedback loops, resulting in their continual release, which perpetuates native tissue damage and pathological ECM deposition and remodeling.

**Table 2:** Macrophage secretions central to fibrotic progression

Pro-inflammatory		
Secreted factor	Function (*Fibroblast* c*rosstalk*)	Source(s)
CCL2	Monocyte chemoattractant protein-1: induces monocyte and fibrocyte trafficking to the fibrotic microenvironment. *Can act as chemoattractant for fibroblasts.*	(72, 177, 178, 179)
CCL3	Macrophage inflammatory protein-1: induces monocyte/macrophage trafficking to the fibrotic microenvironment.	(72, 177, 178, 179)
CCL5	Regulated upon activation, normal T cell expressed and secreted (RANTES): induces monocyte and T cell trafficking to the fibrotic microenvironment.	(72, 177, 178, 179, 180)
TNF-α	Amplifies the immune response in the fibrotic microenvironment by polarizing monocytes into additional macrophages with pro-inflammatory functionality. *Can activate fibroblasts, subsequently increasing production of inflammatory mediators.*	(130, 176, 181, 283)
IL-1β	Amplifies the immune response in the fibrotic microenvironment by polarizing monocytes into additional macrophages with pro-inflammatory functionality. *Can activate fibroblasts, subsequently increasing production of inflammatory mediators.*	(130, 176, 181, 182)
IL-6	Amplifies the immune response in the fibrotic microenvironment by polarizing monocytes into additional macrophages with pro-inflammatory functionality. *Can activate fibroblasts and stimulate myofibroblast differentiation, subsequently increasing ECM production.*	(130, 176, 181, 183, 237)
MMP-2	Gelatinase A: Primarily breaks down gelatin but additionally aids in the breakdown of other ECM proteins including collagens, elastin, fibronectin, and laminin.	(184, 185, 186, 189)
MMP-9	Gelatinase B: Primarily breaks down gelatin but additionally aids in the breakdown of other ECM proteins including collagens, elastin, fibronectin, and laminin.	(184, 186, 189)
MMP-12	Macrophage elastase: Primarily breaks down elastin but additionally aids in the breakdown of other ECM proteins including collagens, fibronectin, and laminin.	(184, 186, 187, 188, 189)
ROS/RNS	Creates oxidative stress, damaging native tissue and further amplifying the immune response. *Can activate fibroblasts, subsequently increasing production of inflammatory mediators.*	(190, 191, 192, 193, 278)

Pro-Reparative		
Secreted factor	Function (*Fibroblast* c*rosstalk*)	Source(s)
CCL17	Thymus and activation-regulated chemokine (TARC): induces T-regulatory cell trafficking to the fibrotic microenvironment.	(195, 196, 197)
CCL22	Macrophage-derived chemokine (MDC): induces T-regulatory and Th2 cell trafficking to the fibrotic microenvironment.	(196)
TGF-β	Induces production of ECM proteins and reprograming to OXPHOS in macrophages. *Can activate fibroblast and stimulate myofibroblast differentiation, subsequently increasing ECM production.*	(157, 158, 229)
IL-10	In the fibrotic microenvironment, its chronic release switches its role from anti-inflammatory functionality to stimulating collagen synthesis and angiogenesis.	(198, 199, 200, 201)
L-ornithine	Produced by ARG-1 activity and acts as a precursor for proline which is a key amino acid in collagen synthesis.	(202, 203, 204)
MMP-1	Collagenase-1: Primarily breaks down collagens but additionally aids in the breakdown of other ECM proteins including, elastin, fibronectin, and laminin.	(184, 185, 186, 189)
MMP-2	Gelatinase A: Primarily breaks down gelatin but additionally aids in the breakdown of other ECM proteins including collagens, elastin, fibronectin, and laminin.	(184, 185, 186, 189)
MMP-12	Macrophage elastase: Primarily breaks down elastin but additionally aids in the breakdown of other ECM proteins including collagens, fibronectin, and laminin.	(184, 186, 187, 188)
TIMPs	Inhibit MMPs to regulate ECM remodeling.	(189, 206, 207)
EGFs	*Enhance myofibroblast contractility.*	(309, 310)

Macrophages responding to pro-inflammatory activation release chemokines and pro-inflammatory cytokines to recruit additional immune cells and amplify the inflammatory response. Chemokines such as CCL-2, -3, and -5 are primarily responsible for the recruitment of additional monocytes to the fibrotic microenvironment (72, 177, 178, 179, 180). Pro-inflammatory cytokines TNF-α, IL-1β, and IL-6, are responsible for the amplification of the immune response by recruiting and differentiating monocytes, while further activating NF-κB signaling and perpetuating the release of additional inflammatory mediations (130, 176, 181, 182, 183). MMPs-2, -9, and -12 are secreted by these macrophages and are responsible for degrading ECM components to ease the infiltration of the additional immune cells (184, 185, 186, 187, 188). The over-release of MMPs destabilizes both newly deposited ECM and the native tissue, which when replaced results in the disorganized arrangement of ECM that exemplifies fibrosis (185, 189). Through the enzymatic activity of NADPH oxidases (NOXs) and inducible nitric oxide synthases (iNOSs), ROS/RNS are produced and secreted, contributing significantly to the dysregulated environment through oxidative stress (190, 191, 192, 193).

The secretome of macrophages responding to pro-reparative activation includes growth factors, chemokines, anti-inflammatory cytokines, MMPs, and TIMPs. TGF-β signaling to other macrophages induces metabolic reprograming, shifting them to oxidative methods of energy production, and stimulates the production of ECM proteins (157, 158). Release of CCL17 and CCL22 recruits additional immune cells, primarily Th2 lymphocytes, that positively reinforce pro-reparative functions (194, 195, 196, 197). Both interactions with TGF-β and Th2 lymphocytes establish a feedback loop in which macrophages are continually exposed to polarizing signals promoting pro-reparative functions, subsequently leading to their over-activation (158, 194, 195). IL-10 is traditionally thought to suppress the production of pro-inflammatory cytokines, but with chronic release, it significantly stimulates collagen synthesis and angiogenesis, contributing to the excessive ECM deposition of fibrosis (198, 199, 200, 201). The enzymatic activity of ARG1 is responsible for the synthesis of L-ornithine, a precursor of proline, a key amino acid in collagen synthesis (202, 203, 204). The high degree of ECM synthesis in macrophages with pro-reparative functionality is pathologic in the fibrotic microenvironment because there is no opportunity for functional tissue to be formed from the ECM (4, 205). MMPs-1, -2, and -12, among others, are secreted to facilitate ECM remodeling (185, 188, 189). However, their function is often inhibited by the simultaneous secretion of TIMPs from the milieu of cells in the fibrotic niche. This imbalance leads to inefficient ECM turnover, allowing damaged and excessive ECM proteins to accumulate (189, 206, 207). The combined effects of excessive ECM synthesis and impaired clearance underpin the fibrotic remodeling process, resulting in tissue stiffness and loss of function.

## Fibroblast signaling is central to fibrotic progression

Fibroblasts encompass a dynamic population dependent on their microenvironmental niche and tissue-specific factors, enabling remarkable versatility in the tissue environment (208, 209, 210). Fibroblasts are typically seen as the effector cells in fibrotic progression due to their ability to extensively proliferate and deposit a large amount of ECM (37, 211, 212). Understanding the lineages, transduction pathways, and secretomes of fibroblasts is critical to characterizing their specialized roles in the pathogenesis of fibrosis. As in the previous section, here we define key fibroblast signaling and its role in the fibrotic-fibroblast pathology; this signaling further contributes to fibrotic progression through cellular crosstalk.

### Fibroblast lineages and functional states in fibrotic disease

Fibroblasts originate from mesenchymal progenitor cells, establishing resident populations that are maintained through proliferation (211). In a fibrotic environment, fibroblast populations can arise from various sources including: primary populations arising due to the increased proliferative efforts of local or migrating adjacent fibroblast resident cells (211, 213), as well as secondary populations arising from epithelial-mesenchymal transition (EMT) (214, 215), pericyte-fibroblast transition (PFT) (216, 217, 218), bone marrow (219, 220, 221), or circulating fibrocytes (222). Broadly, the functional states of fibroblast can be broken down into three main classes: inactive/quiescent fibroblasts, activated fibroblasts, and myofibroblasts (211, 213, 223, 224). Quiescent fibroblasts are primarily involved in the homeostasis of the tissue environment by directing ECM turnover and regeneration in normal physiological conditions (225). In response to the chronic pathological stimuli seen in the fibrotic microenvironment, fibroblasts become activated and/or differentiate into myofibroblasts (40, 212, 223, 226, 227).

Activated fibroblasts and myofibroblasts are induced primarily by the same agent, TGF-β, but exhibit different functionality (215, 223, 228, 229). Outside of TGF-β, these functional states can be induced by stimulation or co-stimulation with fibroblast growth factor (FGF) (230, 231, 232, 233), IL-1 (234, 235, 236), IL-6 (237, 238, 239, 240, 241), and TNF-α (231, 237, 242, 243). Activated fibroblasts are characterized by the surface expression of fibroblast activation proteins (FAPs) (230, 232) and produce large quantities of ECM proteins, predominantly collagens and hyaluronic acid (HA) (212, 213, 223, 233, 244). Continued fibrotic pathogenesis establishes sustained signaling to activated fibroblasts that induces dynamic transitions into myofibroblasts, which are considered the hallmark of fibrosis (223, 245). Myofibroblasts are characterized by high expression of alpha smooth muscle actin (α-SMA) stress fibers, and calponin, allowing for contractile functionality (246, 247, 248, 249, 250). Contracture of the fibrotic ECM causes tissue shortening, increasing stiffness and imparting stiffness-induced mechanical stress of fibrotic effector cells, driving further activation (251, 252). This acts as a positive feedback loop for myofibroblast induction, wherein continually increased mechanical stress acts as a stimulatory agent (128, 252, 253, 254, 255). Outside of their contractile role, myofibroblasts also produce ECM proteins, mainly fibronectin 1 (FN1) and periostin (256, 257, 258, 259, 260). Both activated fibroblasts and myofibroblasts predominately rely on glycolytic methods for energy production, significantly upregulating GLUT1 in response to activation or differentiation (223, 224, 261, 262), although there is considerable diversity in context-specific function. For example, lipofibroblasts that reside in the alveolar interstitium play a specialized role by maintaining a lipid reserve to buffer oxidative stress and releasing FGF10, which promotes alveolar epithelial cell proliferation (263, 264, 265). The complexity of fibroblast functionality is further exacerbated by the emergence of distinctive subclasses that are defined with non-canonical markers such as early B cell factor-1 (EBF1) in fibrotic conditions (266).

### Fibroblast signal transduction pathways implicated in fibrotic progression

The ability of fibroblasts to dynamically transition to different functional states throughout fibrotic disease progression is due to the variety of signal transduction pathways that they exhibit (Table 3). These signal transduction pathways have a high degree of overlap and allow fibroblasts to respond to the various fibroblast-stimulatory agents in the fibrotic microenvironment (212). As in macrophages, the chronic persistence of these stimulatory agents results in over-activation and dysfunction of fibroblast signal transduction (40, 223).

**Table 3:** Fibroblast signal transduction pathways implicated in fibrotic progression

Signalling pathway	Activating ligand	Function	Source(s)
SMAD	TGF-β	Primarily drives the transcription of pro-fibrotic genes.	(156, 157, 161)
YAP/TAZ	Mechanical stress, SMAD,	Promotes the production of ECM proteins (*i.e.*, Collagen I and III) and fibrotic mediators (*e.g.*, TGF-β)	(267, 268, 269, 270, 271)
Notch	Fibroblast contact signals (DLL1/4 and JAG-1/2)	Facilities contact mediated fibroblast activation with the NICD acting as a transcriptional regulator of fibroblast activation genes.	(272, 273, 276, 277)
WNT	β-catenin, Wnt1, Wnt3a	Central to TGF-β signal transduction, leading to the expression of pro-fibrotic genes.	(274, 275)
p38 MAPK	TNF-α, IL-1β, M-CSF, ROS/RNS	Allows fibroblast to response to oxidative stress, upregulating the production of pro-inflammatory cytokines.	(278, 279, 280)
HIF	IL-1β, IL-6, EGF,	Facilitates survival in low oxygen environment and induces pro-inflammatory cytokine release.	(119, 137, 281, 282)
NF-κB	LPS, TNF-α, IL-1β	Induces the production of pro-inflammatory cytokines/chemokines and MMPs	(283, 284, 285)

One of the central signaling pathways to fibroblast functionality is the SMAD pathway. TGF-β induces the phosphorylation of SMAD2 and SMAD3, which complex with SMAD4 and translocate to the nucleus where it drives the transcription of pro-fibrotic genes, namely α-SMA, collagens, and FN1 (156, 157, 161). The SMAD complex can interact with YAP/TAZ complexes allowing SMAD-based transcription to be in response to mechanical stress (267, 268, 269, 270, 271). Outside of this central fibrotic signaling pathway, there are several others that contribute to fibroblast functionality, including Notch, Wnt, HIFs, p38 MAPK, and NF-κB-based signaling. Notch and Wnt signaling both act to further modulate fibroblast-to-fibroblast activation (272, 273, 274, 275). Notch signaling allows for contact-mediated fibroblast activation (276, 277). Upon binding of surface proteins of activated fibroblast/myofibroblasts, the notch receptor intracellular domain (NICD) is cleaved and translocated to the nucleus (277). Once in the nucleus, NICDs act as traditional transcriptional mediators, activating genes involved in fibroblast activation and myofibroblast differentiation (272, 273, 276). Wnt transcriptional intermediates, canonically activated by β-catenin, interact with TCF/LEF factors to drive pro-fibrotic gene expression (274). Wnt signaling is thought to be vital to maintaining proper TGF-β signal transduction (275), and as such its dysregulation is often observed in fibrosis (274).

P38 MAPK, HIF and NF-κB signaling pathways allow fibroblasts to dynamically respond to inflammatory cues in the fibrotic microenvironment. The p38 MAPK pathway allows fibroblasts to respond to oxidative stress and leads to the downstream production of pro-inflammatory cytokines such as IL-6 and TNF-α (278, 279, 280). HIF signaling enables fibroblasts to stabilize their functionality in low oxygen conditions, promoting glycolytic gene transcription. Chronic HIF signaling can cause fibroblasts to exacerbate oxidate stress in the fibrotic microenvironment, further promoting pro-inflammatory cytokine release (119, 137, 281, 282). Lastly, similarly to macrophages, NF-κB signaling is induced by TLRs and IL-1β and drives the expression of pro-inflammatory cytokines, chemokines, and MMPs (283, 284, 285). The chronic activation of these signal transduction pathways perpetuates the production of fibroblast secretomes.

### Fibroblast secretomes central to fibrotic progression

Like macrophages, fibroblasts impart most of their influence on the fibrotic microenvironment through their secretory profiles. The secretome of fibroblasts includes ECM proteins that make up the fibrous tissue and a vast array of cytokines, chemokines, growth factors, and MMPs that define their autocrine/paracrine signaling that modulate the functionality of the milieu of cells in the fibrotic niche (Table 4). In the fibrotic microenvironment, chronic activation skews this network toward excessive ECM production, unrestrained inflammation, and impaired resolution, establishing a self-reinforcing loop that drives disease progression (4, 40, 224).

**Table 4:** Fibroblast secretions central to fibrotic progression

Signalling pathway	Function (*Macrophage crosstalk*)	Source(s)
Collagen I	Major structural component of fibrotic ECM. Primarily responsible or ECM stiffness.	(286, 287, 288)
Collagen III	Major structural component of fibrotic ECM. Less prevalent than collagen I, and is responsible, along with elastin for elastic rebound of fibrotic tissue.	(286, 287, 288)
FN1	Major structural component in fibrotic ECM. Acts as a scaffold for new ECM deposition sites, enabling the formation of cross-linked networks that increase ECM stiffness.	(286, 287, 288, 289, 290, 291)
HAs	Minor structural component in fibrotic ECM.	(286, 287, 288)
Elastin	Minor structural component in fibrotic ECM. Responsible for elastic rebound of fibrotic tissue.	(286, 287, 288)
Proteoglycans	Minor structural component in fibrotic ECM.	(286, 287, 288)
MMP-1	Collagenase-1: Primarily breaks down collagens but additionally aids in the breakdown of other ECM proteins including, elastin, fibronectin, and laminin	(184, 185, 186, 189, 252)
MMP-2	Gelatinase A: Primarily breaks down gelatin but additionally aids in the breakdown of other ECM proteins including collagens, elastin, fibronectin, and laminin.	(184, 185, 186, 189, 252)
MMP-3	Stromelysin-1: Aids in the breakdown of ECM proteins including collagens, proteoglycans, fibronectin, elastin, and laminin.	(189, 252)
MMP-9	Gelatinase B: Primarily breaks down gelatin but additionally aids in the breakdown of other ECM proteins including collagens, elastin, fibronectin, and laminin.	(184, 186, 189, 252)
MMP-13	Collagenase 3: Primarily breaks down collagens but additionally aids in the breakdown of other ECM proteins including, elastin, fibronectin, and laminin	(189, 252)
TIMPs	Inhibit MMPs to regulate ECM remodeling.	(189, 206, 207)
TGF-β	Induces fibroblast activation, proliferation, and myofibroblast differentiation. *Induces production of ECM proteins and reprograming to OXPHOS in macrophages.*	(157, 158, 160, 229)
FGF	Induces fibroblast activation and proliferation.	(292, 293)
PDGFs	Stimulates ECM production through MAPK signalling. Induces fibroblast migration to the fibrotic microenvironment and promotes proliferation. *Can be secreted by macrophages.*	(294, 295, 306)
VEGFs	Promotes angiogenesis to counteract hypoxia of the fibrotic microenvironment. Induces fibroblast migration to the fibrotic microenvironment and promotes proliferation as well as myofibroblast differentiation. *Can be secreted by macrophages.*	(296, 307)
CTGFs	Promotes myofibroblast differentiation. Induces fibroblast migration to the fibrotic microenvironment and promotes proliferation. *Can be secreted by macrophages.*	(297, 308)
IL-1β	Stimulates fibroblasts to release pro-inflammatory mediators. *Induces pro-inflammatory functionality in macrophages.*	(181, 182, 237)
IL-6	Stimulates fibroblasts to release pro-inflammatory mediators. *Induces pro-inflammatory functionality in macrophages.*	(181, 183, 237)
TNFα	Stimulates fibroblasts to release pro-inflammatory mediators. *Induces pro-inflammatory functionality in macrophages.*	(181, 278, 280)
M-CSF/GM-CSF	*Promotes macrophage survival and differentiation.*	(68, 302, 303, 304)

ECM itself forms a significant portion of the fibroblast secretome. Activated fibroblasts and myofibroblasts produce large quantities of ECM components: collagens (predominantly collagen I and III), HAs, elastin, laminins, and proteoglycans that maintain the structural integrity and elasticity of the fibrotic ECM (286, 287, 288). Myofibroblasts additionally upregulate the production of fibronectins, mainly FN1. Fibronectin-enriched ECM serves as a scaffold for additional ECM deposition sites, enabling the formation of densely cross-linked networks that dramatically increase ECM stiffness (288, 289, 290, 291). This increases mechanical stress, significantly contributing to the aforementioned positive feedback loop to fibroblast activation (252, 254, 267). In response to this mechanical stress, fibroblasts upregulate the production of MMPs, primarily MMP-1, -2, -3, -9, and -13 (252), which facilitates aggressive ECM degradation that is dysfunctional due to the high level of TIMPs in the microenvironment. Ultimately, the imbalance in MMPs and TIMPs results in damaged ECM accumulation due to improper cleavage, subsequently myofibroblasts preferentially bind to these damaged regions and contract the ECM, resulting in increased ECM density and further adding to the mechanical stress of the environment (189).

Growth factor production plays a central role in the fibroblast secretome. Fibroblasts secrete growth factors including TGF-β, FGFs, platelet-derived growth factors (PDGFs), VEGFs, and connective tissue growth factors (CTGF). Both TGF-β and FGFs amplify the fibroblast response *via* an autocrine signaling loop (158, 160, 229, 292, 293). PDGFs, VEGFs, and CTGFs all act to further stimulate fibroblast proliferation rate and promote fibroblast migration from tissues adjacent to the primary fibrotic environment (294, 295, 296, 297). In addition to this role, PDGFs also stimulate ECM production *via* MAPK signaling (298). Angiogenic factors, such as VEGFs, direct the formation of new blood vessels that can counteract the hypoxic nature of the fibrotic microenvironment (114, 299). Additionally, both VEGFs and CTGFs can help to induce myofibroblast differentiation (300, 301). Outside of these growth factors, fibroblasts can also produce pro-inflammatory cytokines, namely, IL-1β and IL6, facilitated through the activation of NF-κB (237). This enables fibroblasts to contribute to the perpetuation of inflammation in the fibrotic microenvironment.

## Macrophage–fibroblast crosstalk in fibrotic microenvironments

**Figure 2:**
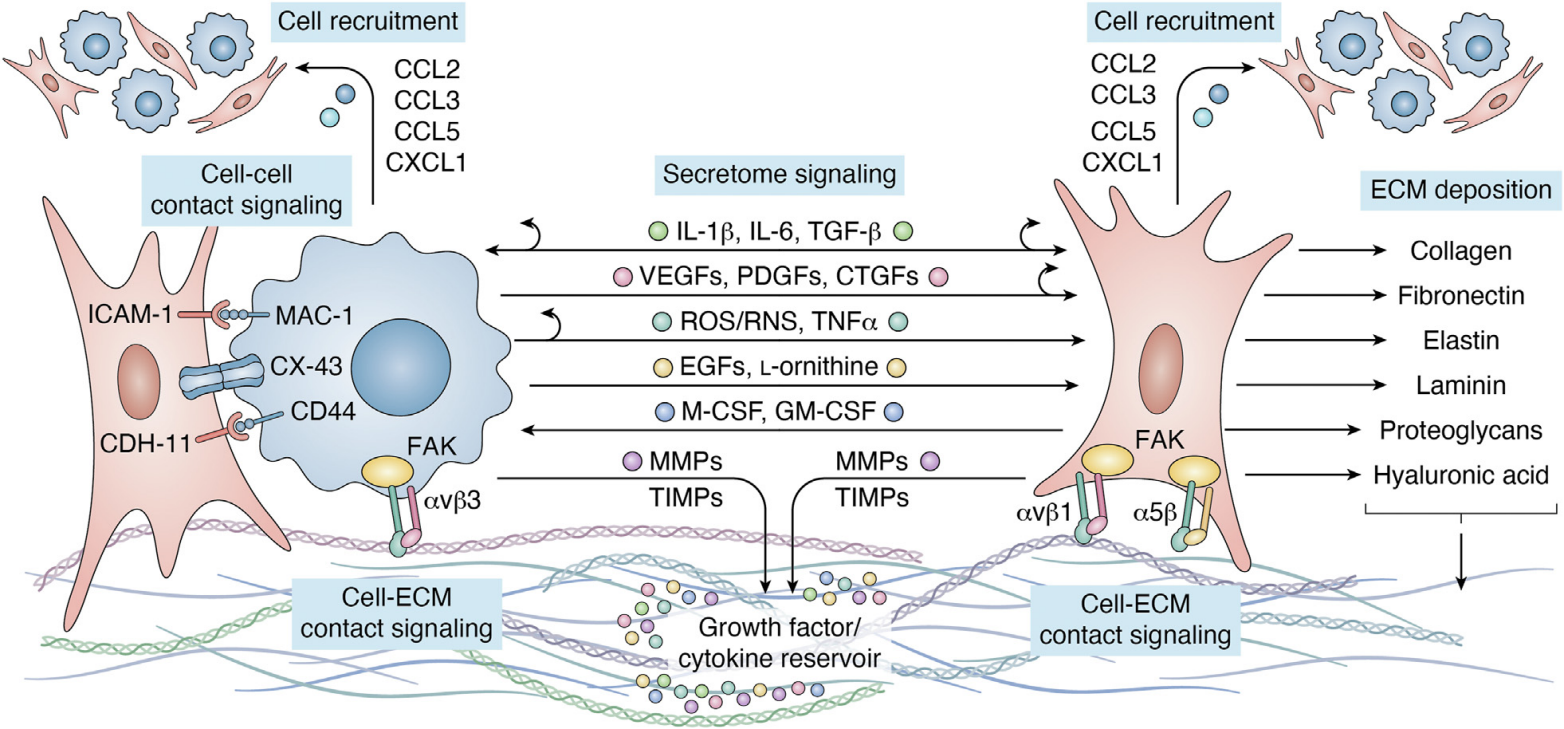
**Dynamic macrophage–fibroblast reciprocal interactions.** Macrophages and fibroblasts primarily communicate (recruiting additional cells or activating existing cells) through secretome signaling that can act either on the opposite (→, ↔) or on the same cell type (↻) (potentially in an autocrine fashion). Additionally, macrophage and fibroblast both secrete MMPs (balanced by TIMPs) that degrade the ECM, potentially releasing trapped inducing factors (growth factors/cytokines). These cells further activate one another through cell-cell contact signaling. These cells also exhibit contact signaling with the ECM, which can further modulate their activation. Fibroblast activation drives ECM deposition, which further adds onto dynamic interactions through cell attachment and diffusion limitations of activating factors.

Macrophage–fibroblast crosstalk is a dynamic and multifaceted process that underscores the pathogenesis of fibrosis. Bidirectional interactions establish a chronic positive feedback loop that perpetuates and sustains the pathological functional states demonstrated in the milieu of cells in the fibrotic microenvironment. These feedback loops are driven by the reciprocal exchange of fibrotic and inflammatory mediators, such as IL-6 and TGF-β, that reinforce the functional states of both macrophage and fibroblasts and ensure a continuous supply of ECM components (41, 160, 237). The bidirectional nature of these secretomes enforces the chronic unresolving inflammatory environment that underscores pathological fibrosis (Fig. 2) (37).

One of the most significant signaling axes in macrophage-fibroblast interactions is the M-CSF/granulocyte-macrophage colony-stimulating factor (GM-CSF) transduction pathway. Activated fibroblasts secrete M-CSF and GM-CSF that binds to M-CSF/GM-CSF receptors on macrophages, promoting survival and differentiation. This interaction often skews macrophage polarization towards pro-fibrotic functionality, upregulating the production of pro-fibrotic signaling factors (68, 302, 303, 304, 305). The most studied pro-fibrotic factor is TGF-β, which is produced by both activated macrophages and fibroblasts. TGF-β, through engagement with TGF-β receptors, promotes fibroblast activation, myofibroblast differentiation, and macrophage activation toward pro-fibrotic functionally (158, 160, 229). Other pro-fibrotic factors such as VEGFs, PDGFs, and CTGFs are released by both macrophages and fibroblasts. These factors primarily act to amplify the fibroblastic activity within the fibrotic microenvironment by impacting fibroblast migration, proliferation, and ECM deposition (114, 294, 295, 300, 306, 307, 308). Furthermore, macrophages express epidermal growth factors (EGFs), primarily amphiregulin, in response to tissue damage, which further exacerbate myofibroblast contractility (309, 310).

The bidirectional nature of macrophage–fibroblast crosstalk is further exemplified through IL-1β and IL-6 secretion from both macrophages and fibroblasts. Secreted IL-1β or IL-6 can act in either an autocrine or paracrine fashion to perpetuates cellular activation and inflammation (37, 41, 235, 237). Another crucial group of secreted fibrotic mediators are CCL and CXCL chemokines. Macrophages and fibroblasts secrete CCL2, CCL5, and CXCL1 which recruit additional pro-inflammatory macrophages and other immune cells to the fibrotic environment (178, 180, 198, 311, 312, 313, 314, 315). CCL2 has also been identified as a chemoattractant for fibroblasts (316). Conversely to the attractive effects of these chemokines, macrophage-produced CXC10 has been identified to inhibit growth-factor-induced fibroblast migration (315, 317). Beyond soluble factors, macrophages and fibroblasts engage in direct contact-mediated signaling through receptor-ligand interactions. Intercellular adhesion molecule-1 (ICAM-1) on fibroblasts binds to macrophage-1 antigen (MAC-1) on macrophages, facilitating adhesion and promoting the activation of both cell types (318, 319). Similarly, cadherin-11 (CDH-11) on fibroblasts binds to CD44 on macrophages, promoting fibroblast activation *via* increased Wnt signaling (320, 321, 322). Finally, syncytial signaling through connexin-43 (CX-43) represents another layer of macrophage-fibroblast contact interaction by enabling direct cytoplasmic communication, synchronizing cellular responses to the fibrotic environment (323, 324, 325).

### ECM as a modulator of macrophage–fibroblast interactions

ECM is not merely a structural scaffold but an active participant in macrophage–fibroblast crosstalk; matrix composition and mechanical properties influences cellular behavior, signaling, and functional population plasticity (326, 327, 328). Integrins serve as critical mediators of cell–ECM interactions (328, 329). Macrophages express integrin αvβ3, which binds to fibronectin, promoting their polarization to the pro-fibrotic functionalities (120). Conversely, fibroblasts express integrins such as αvβ1 and α5β, which engage with ECM components such as collagen to drive their activation and ECM synthesis (330, 331). ECM stiffness and mechanical tension further modulate cellular responses through focal adhesion kinase (FAK) signaling (332, 333). Both macrophages and fibroblasts express FAK, which integrates mechanical cues from ECM into their intracellular signaling cascades (334). This mechanotransduction enhances cellular activation and perpetuates ECM remodeling (255, 271, 332). ECM also serves as a reservoir for cytokines and growth factors, modulating their bioavailability and activity (335). For instance, TGF-β stored in ECM can be activated through mechanical stress or proteolytic cleavage, ensuring its sustained activity within the fibrotic niche (336).

## Macrophage–fibroblast dynamics in late-stage pathologies

**Figure 3:**
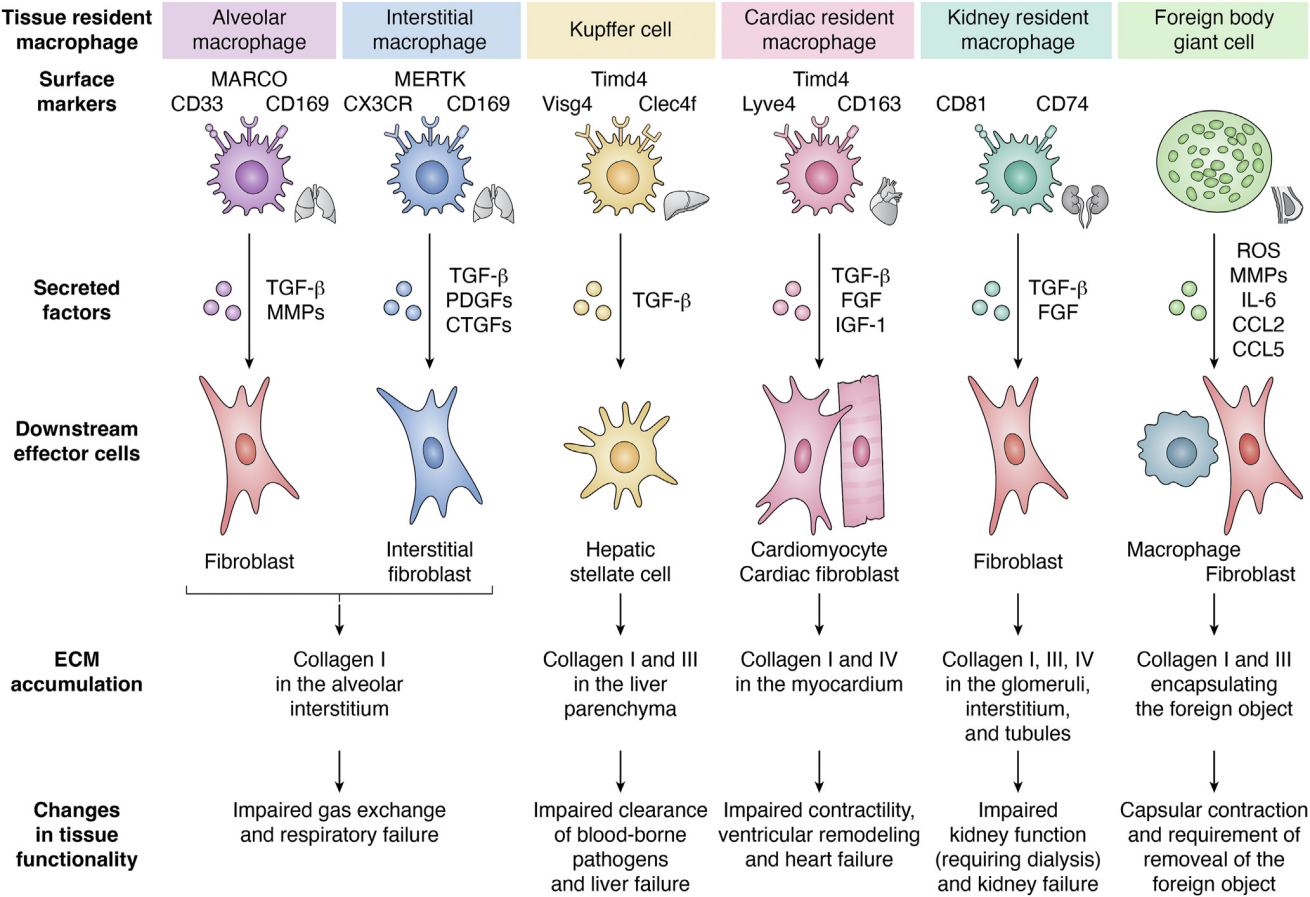
**Resident macrophages in fibrotic pathologies.** Tissue resident macrophages exhibit unique interactions in fibrotic disease progression dependent on their specific tissue microenvironment and tissue resident stromal cells. The fibrotic ECM profile varies in each pathology, with the most notable differences being in the dominate collagen types. Ultimately, the loss of tissue resident macrophages plays a key role in the downstream tissue pathology.

ECM remodeling is a critical component in the progression of fibrosis across various tissues, playing a central role in fibrotic disease progression. This process involves the accumulation of ECM proteins that impair the tissue’s normal architecture, leading to irreversible tissue damage, organ dysfunction, and eventual failure. With all pathological fibrotic conditions, macrophages are pivotal in driving fibrotic pathogenesis through their lineage-specific contributions to fibrotic progression and cellular crosstalk with fibroblast cells (Fig. 3).

### Pulmonary fibrosis

Idiopathic pulmonary fibrosis (IPF) is a chronic, progressive interstitial lung disease characterized by the excessive deposition of ECM components, leading to the destruction of lung architecture, impaired gas exchange, and respiratory failure (11, 12, 14). Without obvious etiology, IPF likely results from repetitive micro-injuries, such as aspiration, viral infection, or mechanical stress, acting on a genetically, epigenetically, and possibly environmentally (*e.g.*, smoking) primed lung environment. These insults provoke aberrant wound healing responses that unfold over time, evolving from localized inflammation to regionally entrenched fibrosis. IPF is characterized by the accumulation of collagen, particularly type I collagen, in the alveolar interstitium (10, 14). This is a spatially defined disease that originates primarily from the interstitial space with impact on local conducting zones and alveoli; other nearby conducting zones or neighboring interstitial space can be unimpacted despite a high degree of pathology in the concerned region (337). At baseline, tissue-resident alveolar macrophages (AMs) are responsible for the clearance of air-borne pathogens and particulate matter and drive mucus production during lung infections (54, 56, 338). Concurrently, tissue-resident interstitial macrophages (IMs) maintain interstitial homeostasis and produce high levels of IL-10 to combat allergic responses to inhaled allergens (339).

Early IPF is marked by transient epithelial stress and immune cell infiltration, which initially may attempt tissue repair (12). However, alveolar type II cells, central to epithelial homeostasis, exhibit impaired regenerative capacity. This leads to a breakdown in epithelial integrity and promotes pro-fibrotic signaling (340, 341). Concurrently, fibroblasts begin to secrete a suite of factors including TGF-β, PDGF, and VEGF, which not only activate neighboring fibroblasts but also recruit and polarize macrophages toward pro-fibrotic phenotypes (114, 229, 241, 306, 337). Activated fibroblasts also exhibit resistance to apoptosis and adopt a highly secretory functionality further increasing their secretion of TGF-β, PDGF, and VEGF and additionally secreting MMPs and ROS that perpetuate tissue damage and immune cell infiltration (266, 288, 342, 343). In parallel, infiltrating macrophages flood the fibrotic microenvironment and adopt acute pro-inflammatory functionalities; AM populations significantly increase alongside infiltrating macrophage populations (56). These cells, together with IM populations, modulate and mediate acute inflammation through release of pro-inflammatory cytokines and dysregulated inflammasome activity, particularly through the NLRP3 pathway (57, 196, 344, 345). As such, chronic IL-1β signaling is implicated in IPF and leads to impaired tissue repair responses (345, 346).

In later stages of IPF, the reprogrammed highly secretory activated fibroblasts become autonomous and regionally persistent, inducing a shift in macrophage populations to pro-fibrotic functionalities primarily *via* sustained TGF-β signaling. Pro-fibrotic infiltrating macrophage and AM populations contribute more heavily to dysregulated ECM remodeling through secretion of MMPs and further promote fibroblast activation *via* cytokines such as IL-1β, TNF-α, and TGF-β (56, 231, 339). IMs interact directly with interstitial fibroblasts through juxtracrine TGF-β signaling, inducing their differentiation and stimulating production of TGF-β to propagate myofibroblast transitions (57, 339, 347). Furthermore, IMs secrete PDGFs and CTGFs to help stabilize fibroblast populations (57). These pro-fibrotic functional states are further exacerbated by recurrent secondary injuries, such as infection, aspiration, and alveolar collapse, which can locally accelerate fibrosis even in the absence of new initiating signals (10, 348). In addition, protective regulators such as Nrf2, which mitigate oxidative stress and TGF-β-driven fibroblast activation, appear overwhelmed or functionally suppressed in progressive IPF disease (349). These microenvironmental shifts, layered onto a background of impaired epithelial repair and innate immune dysfunction, help explain the spatial and temporal heterogeneity observed in IPF lungs and underscore the irreversible nature of late-stage fibrosis.

### Hepatic fibrosis

Liver fibrosis is a common consequence of chronic liver injury due to factors such as viral infections (*e.g.*, hepatitis B or C), alcohol use, or metabolically associated steatohepatitis (MASH). In liver fibrosis, there is excessive deposition of ECM proteins, particularly collagen types I and III, in the liver parenchyma. This leads to the impaired clearance of blood-borne pathogens and development of cirrhosis, portal hypertension, and, ultimately, liver failure (15, 16). Tissue-resident Kupffer cells (KCs) primarily reside within the liver sinusoids (61). While in the sinusoids, KCs act as the body’s microbial filter for blood-borne pathogens. KCs significantly contribute to fibrotic disease progression by inducing hepatic stellate cells (HSCs; the resident fibroblasts of the liver) to transition to myofibroblasts (59, 61, 350). KCs interact directly with HSCs *via* pseudopodia while in the sinusoids, allowing juxtracrine TGF-β signaling (59). The alteration of the liver parenchyma tissue architecture *via* the accumulation of collagen types I and III results in the loss sinusoids and subsequently causing circulation to be redirected to through high-flow collateral vessels (351). As a result, blood-borne pathogens are no longer filtered out by KCs. In compensation, infiltrating macrophages seed the larger vessels to form a KC-like syncytium with heightened capacity to capture pathogens, albeit with less success due to the higher flow rates in the collateral vessels (351).

### Renal fibrosis

Kidney fibrosis is a common feature of chronic kidney disease (CKD), glomerulosclerosis, and tubular atrophy, which ultimately leads to end-stage renal disease (ESRD) and the need for dialysis or kidney transplantation. Kidney fibrosis is characterized by the excessive deposition of ECM, primarily collagen types I, III, and IV, in the glomeruli, interstitium, and tubules, leading to impaired renal function (18, 19, 352). The current understanding of the role of kidney resident macrophages (KRMs) in the fibrotic pathogenesis is limited to what is known for all tissue resident macrophages; KRMs are responsible for the establishing the acute inflammatory environment that leads to the recruitment of infiltrating macrophages (63, 131, 132). As the fibrotic microenvironment progresses, KRMs adopt a more pro-fibrotic role, secreting signaling factors such as TGF-β and FGF to stabilize fibroblast populations and promote myofibroblast differentiation (132, 353, 354).

### Cardiac fibrosis

Cardiac fibrosis is a major contributor to the progression of heart disease, particularly after myocardial infarction (MI), with chronic hypertension, and in the development of diastolic heart failure (7). Cardiac fibrosis is characterized by excessive deposition of ECM components, particularly collagen types I and IV, in the myocardium, causing pressure-overload. Cardiac fibrosis can be spatially limited to specific conducting zones, affecting the cardiac rhythm (355). This leads to increased ventricular remodeling, impaired contractility, reduced cardiac function and, ultimately, heart failure with preserved ejection fraction (HFpEF) (7). Cardiac resident macrophages (CRMs) form a distinctive network with cardiomyocytes (CMs) *via* connexin-43, allowing juxtracrine signaling of growth factors, such as insulin-like growth factor 1 (IGF-1), to support cardiomyocyte growth and help regulate electrical conductivity (67). Additionally, CRMs are responsible for the clearance of apoptotic and senescent cells, further contributing to cardiac homeostasis (66, 67). In the fibrotic microenvironment, CRMs have been identified to accelerate cardiomyocyte hypertrophy and death through TNF-α signaling (66). CRMs also signal to cardiac fibroblasts with TGF-β to induce myofibroblast differentiation (356). However, CRMs have also been demonstrated to play a protective role against fibrosis and subsequent impaired cardiac functions. In response to pressure overload, CRMs secrete increased amounts of anti-inflammatory cytokines, such as IL-10, and stimulate the microvascular growth in increased VEGF signaling (65). Both actions help to alleviate the increased pressure in the fibrotic heart. Furthermore, a distinctive subpopulation of CRMs has been identified, characterized by a high expression of MHC-II, that restricts the entry of infiltrating monocyte (65, 66).

### Implant-induced fibrosis

Implant-induced fibrosis refers to the fibrotic response generated by the implantation of foreign materials, such as medical devices, prosthetics, or non-medical persistent foreign materials. This foreign body response (FBR) leads to the formation of a fibrous capsule around the implanted material, impairing its function and leading to complications (357, 358); low levels of fibrotic encapsulation induced by the FBR is necessary for most cases of implant–host tissue integration. The FBR is characterized by the adsorption of host proteins to the foreign material, functioning as constitutive activating factors (357). The persistence of these activating signals, particularly pro-phagocytotic and IL-4/IL-13, frustrates macrophages in the microenvironment and subsequently results in the fusion of these macrophages into multinucleated foreign body giant cells (FBGCs) (359, 360). Despite being considered a hallmark of the FBR, little is known about their exact mechanistic role in fibrotic pathogenesis. FBGCs are thought to have very similar, but heightened, signaling roles as traditional macrophages with the release of pro-inflammatory cytokines and chemokines, such as IL-6, CCL2, and CCL5 (359, 361). In additional to these more traditional roles, FBGCs have increased phagocytic ability due to their increased size (up to 1 mm in diameter) (359) and priming of absorbed proteins to foreign object surfaces through complement opsonization (359, 362). Furthermore, FBCGs have been shown to strongly adhere to the foreign material surface, generating and isolated extracellular environment that acts as an extracellular lysosome through the increased secretion of ROS and MMPs into the environment (359, 363, 364). FBGCs have not been identified to have direct communication with fibroblasts (38). Instead, in the FBR fibroblast functionality is primarily in response to the abrupt and marked increase stiffness gradient relative to the native tissue environment due to the presence of the foreign material (2, 38, 365).

## Conclusion and outlook

The current understanding of macrophage–fibroblast cellular crosstalk summarized in this review indicates complex cellular population plasticity and dynamic behaviors. Macrophages and fibroblasts are central players in the pathogenesis of fibrosis, with their interactions driving the progression and severity of fibrotic diseases. These cell types engage in dynamic and reciprocal crosstalk that fosters the accumulation of ECM components, tissue remodeling, and the perpetuation of pathological fibrotic environments through persistent inflammatory signaling. Through the bidirectional exchange of soluble factors and direct cell-to-cell interactions, macrophages and fibroblasts work in concert to activate and sustain each other's fibrotic roles, creating a positive feedback loop that exacerbates the fibrotic process. This mutual stimulation and continuous interaction reinforce the milieu of cells within the fibrotic niche, ensuring the persistence of fibrosis across various organs.

Ultimately, this review summarizes the current understanding of the dynamically adapting macrophage–fibroblast reciprocal interactions that are central to the pathogenesis of all fibrotic pathologies. Additionally, we consolidate recent findings concerning the roles of tissue-resident macrophage and stromal cells in the development of fibrosis within their respective environments. Importantly, we stress the modularity of macrophage functions with respect to canonical classification, highlighting the importance of functional characterization of macrophages in the fibrotic milieu.

Understanding the tissue-specific dynamics of macrophage-fibroblast interactions is essential to comprehending the pathophysiology of fibrosis. In each tissue, macrophages adopt roles that contribute to fibroblast activation, ECM deposition, and the progression of fibrosis. These interactions, driven by a combination of soluble factors and ECM-specific mechanical cues, foster the chronic inflammation and loss of tissue architecture and function that characterize fibrotic diseases. The specific contributions of macrophages and fibroblasts, influenced by the local tissue environment, underscore the complexity of fibrosis and highlight the importance of considering these interactions in therapeutic strategies. Full understanding of tissue-specific environmental cues and context-dependent mechanisms of fibrosis is currently lacking, impeding clinical translation of new therapeutic candidates. This is further exacerbated by the lack of preclinical models that capture the heterogeneity of fibrotic diseases in clinical settings, and emerging data that suggests distal tissues environmental cues or pathologies can reprogram infiltrating cells to have enhanced pro-fibrotic capacity. For example, cardiac fibrosis is increased post-stroke and pulmonary fibrosis is increased in aged populations due to IL-1-mediated (366) and aging-induced reprogramming of developing monocytes (367). These findings suggest that systemic factors can influence the clinical presentation of tissue-specific fibrotic disease, further complicating both mechanistic study and the development of treatments.

Tissue-specific understanding of pathological fibrosis has been fundamentally accelerated with the use of high-parameter analyses such as scRNAseq, spatial transcriptomics, and other omics methodologies. These methods enable higher resolution analysis of specific environmental cues by defining exact macrophage and fibroblast population functionalities through examining their entire expression profiles. For example, temporal spatial transcriptomics of pulmonary fibrosis has revealed a distinctive dysregulated cellular niche that precedes changes to tissue architecture. Within this niche TGF-β signaling has been demonstrated to be central to the disease progress (337, 342). In addition to these high-parameter analyze methods, tissue-specific lineage tracing would vastly improve our understanding of which cell populations are driving fibrotic progression. Improved knowledge of tissue-specific macrophage-fibroblast reciprocal interaction would allow for new tissue-specific therapeutic targets to be discovered and utilized in the development of novel anti-fibrotic medications. Importantly, the multivariate etiology of pathological fibrotic diseases makes the use of anti-fibrotic therapies that target only one implicated biological pathway often ineffective. Therefore, experiments may benefit from exploration of synergistic anti-fibrotic strategies by targeting multiple implicated pathways.

## Conflict of interest

The authors declare that they have no conflicts of interest with the contents of this article.
